# Pelvic exenteration for colorectal and non-colorectal cancer: a comparison of perioperative and oncological outcome

**DOI:** 10.1007/s00384-021-03893-y

**Published:** 2021-03-07

**Authors:** Andreas Bogner, Johannes Fritzmann, Benjamin Müssle, Johannes Huber, Jakob Dobroschke, Ulrich Bork, Steffen Wolk, Marius Distler, Jürgen Weitz, Thilo Welsch, Christoph Kahlert

**Affiliations:** 1 Department of Visceral, Thoracic and Vascular Surgery, Faculty of Medicine Carl Gustav Carus, Technische Universität Dresden, Fetscherstrasse 74, 01307 Dresden, Germany; 2 Department of Urology, Faculty of Medicine Carl Gustav Carus, Technische Universität Dresden, Dresden, Germany; 3 Department of General, Visceral, Thoracic and Proctologic Surgery, Helios Klinikum Pirna, Pirna, Germany

**Keywords:** Pelvic exenteration, Rectal cancer, Recurrence, Survival, Surgical complication

## Abstract

**Background:**

Pelvic exenteration (PE) is the only option for long-term cure of advanced cancer originating from different types of tumor or recurrent disease in the lower pelvis. The aim was to show differences between colorectal and non-colorectal cancer in survival and postoperative morbidity.

**Methods:**

Retrospective data of 63 patients treated with total pelvic exenteration between 2013 and 2018 are reported. Pre-, intra-, and postoperative parameters, survival data, and risk factors for complications were analyzed.

**Results:**

A total of 57.2% (*n* = 37) of the patients had colorectal cancer, 22.3% had gynecological malignancies (vulvar (*n* = 6) or cervical (*n* = 8) cancer), 11.1% (*n* = 7) had anal cancer, and 9.5% had other primary tumors. A total of 30.2% (*n* = 19) underwent PE for a primary tumor and 69.8% (*n* = 44) for recurrent cancer. The 30-day in-hospital mortality was 0%. Neoadjuvant treatment was administered to 65.1% (*n* = 41) of the patients and correlated significantly with postoperative complications (odds ratio 4.441; 95% CI: 1.375–14.342, *P* > 0.05). R0, R1, R2, and Rx resections were achieved in 65.1%, 19%, 1.6%, and 14.3% of the patients, respectively. In patients undergoing R0 resection, 2-year OS and RFS were 73.2% and 52.4%, respectively. Resection status was a significant risk factor for recurrence-free and overall survival (OS) in univariate analysis. Multivariate analysis revealed age (*P* = 0.021), ASA ≥ 3 (*P* = 0.005), high blood loss (*P* = 0.028), low preoperative hemoglobin level (*P* < 0.001), nodal positivity (*P* < 0.001), and surgical complications (*P* = 0.003) as independent risk factors for OS.

**Conclusion:**

Pelvic exenteration is a procedure with high morbidity rates but remains the only curative option for advanced or recurrent colorectal and non-colorectal cancer in the pelvis.

**Supplementary Information:**

The online version contains supplementary material available at 10.1007/s00384-021-03893-y.

## Introduction

Alexander Brunschwig described the first total pelvic exenteration (PE) in 1948. Since then, the procedure has evolved significantly in the last decades [1]. Initially, PE was mainly performed in palliative intent in patients with recurrent cervical cancer. Today, colorectal cancer and locally recurrent rectal cancer account for the majority of cases due to the successful introduction of multimodal therapy concepts [2]. In addition, PE is often performed on patients with locally progressive tumors of gynecological origin (cervical or vulvar/vaginal cancer), anal squamous cell carcinoma, or sarcomas [3, 4]. In many of these cases, PE represents the only remaining chance of a complete cure. However, this comes at the price of significant perioperative morbidity. Despite an acceptable mortality rate (~2–5%), complication rates of up to 86% [5] have been reported. These rates were associated with longer hospitalization, resulting in a lengthy rehabilitation period until the patients were able to return to an acceptable performance status [5]. Despite the high risk of complications, the indication to perform a PE is often justified because non-resection of the tumor may also result in great loss of quality of life (e.g., a superinfected necrotic tumor in the small pelvis or tumor-associated fistulas). However, there should be a high probability of obtaining an R0 resection when planning the operation. R0 resection is one of the main factors associated with long-term survival in patients undergoing PE for colorectal and non-colorectal cancer [6, 7]. Achieving an R0 resection can often be technically demanding, especially if the tumor extends to the lateral pelvic compartment [8]. The challenge for the surgeon is to operate as radical as necessary while preserving as much function and tissue as possible, especially nerves and bone. Experienced surgeons can achieve a tumor-free resection margin in up to 80% of cases in primary tumors and 40–70% of cases in recurrent disease [9–11].

Most studies on PE have focused exclusively on either (colo) rectal cancer or non-colorectal cancer. So far, there have been only a few studies comparing whether tumor or histological type has a different impact on short- and long-term morbidity and mortality. We therefore included all consecutive patients in this study who underwent PE in our department between January 2013 and May 2018. The aim of this study was to determine whether perioperative outcome and long-term survival differed significantly between patients with colorectal cancer and non-colorectal tumors.

## Patients and methods

This study was approved by the local institutional review board and conducted in compliance with the Declaration of Helsinki, according to the ICH Harmonized Tripartite Guideline for Good Clinical Practice.

### Preoperative assessment

The results of all patients treated with pelvic exenteration from January 2013 to May 2018 in our university hospital were reported. Only patients with locally advanced malignant diseases were included in this retrospective analysis. All patients were presented to an interdisciplinary tumor board preoperatively. All patients signed a written informed consent. Tumors were confirmed histologically. Preoperative imaging was available for all patients (CT scan of the thorax, abdomen, and pelvis; MRI of the pelvis).

### Perioperative setting and follow-up

The operational procedure included radical en bloc resection of all pelvic organs, including the rectum, sigmoid colon, distal ureters, bladder, internal reproductive organs (prostate, seminal vesicles, uterus, vagina, and cervix), surrounding lymph nodes, and pelvic peritoneum. If necessary, adherent (muscle, ligament, neurovascular, or bone) tissue was resected in order to achieve an R0 situation. All operations were performed or supervised by an experienced abdominal/colorectal surgeon. Urinary reconstruction was performed by the urology department using a standard ileal conduit [12]. Preoperative blood samples were collected from all patients. All patients were treated according to early recovery after surgery principles [13]. Clinical data were obtained from the internal documentation system and follow-up data were collected during patient visits to our outpatient clinics or in telephone interviews with their general practitioners or oncologists.

### Morbidity and mortality

Perioperative complications were recorded and classified according to Dindo et al. [14]. A grade ≥ 3b was considered a need for reoperation and thus a major complication. Mortality was defined as the occurrence of death within 30 or 90 days after surgery.

### Statistics

Due to the retrospective character of the reported data, sample size was not chosen based on a power calculation. For the surgical complication analysis, categorical variables were compared using the *χ*^*2*^ test. Continuous variables were expressed as median and interquartile range (IQR) and compared using Student’s *t* test or the Wilcoxon rank-sum test. All variables with *P* < 0.1 were included in a stepwise backward multivariate logistic regression model using the median as a cutoff and adjusting for age, sex, BMI, and ASA. Results were reported as odds ratios (OR) and 95% confidence intervals (95% CI). Overall survival (OS), defined as time to death, and recurrence-free survival (RFS), defined as time to recurrence, were determined. Kaplan–Meier curves were obtained to visualize the differences in OS and RFS. Univariate testing was performed using the log-rank test. All univariate analyses with *P* < 0.05 and non-dichotomized continuous variables were added to a multivariate Cox regression model adjusting for age, sex, BMI, and ASA. No adjustment was made for multiple testing. A *P* value < 0.05 was considered statistically significant. Results were reported as hazard ratios (HR) and 95% confidence intervals (95% CI). Statistical analyses were carried out using IBM SPSS Statistics v23 (SPSS Inc., Chicago, IL) and GraphPad Prism v7 (GraphPad Software, Inc., La Jolla, CA) for graphical illustration.

## Results

### Patient characteristics

Between January 2013 and May 2018, a total of 63 consecutive patients underwent a total pelvic exenteration with a median follow-up time of 19.4 months (IQR 10.0–32.9) (Table 1). In total, 57.2% (*n* = 37) of the patients had colorectal cancer, 22.3% had gynecological malignancies (vulvar (*n* = 6) or cervical (*n* = 8) cancer), 11.1% (*n* = 7) had anal cancer, and 9.5% had other primary tumors (prostate, sarcoma). A total of 30.2% (*n* = 19) of the patients underwent PE for a primary tumor and 69.8% (*n* = 44 patients) for recurrent cancer. The median length of stay was 43.0 (IQR 25.0–79.0) days in the hospital and 4.0 (IQR 3.0–6.0) days in the intensive care unit.

**Table 1:** Patients’ characteristics

	No. (%)	Median (IQR)
Preoperative data		
Age	63	61.7 (53.8-69.9)
< 65 years	42 (66.7)	
> 65 years	21 (33.3)	
Gender		
Male	31 (49.2)	
Female	32 (50.8)	
BMI (kg/m^2^)	63	24.2 (21.7-26.9)
ASA score		
I	2 (3.2)	
II	18 (28.6)	
III	43 (68.3)	
Diagnosis		
Rectal cancer	36 (55.6)	
Colonic cancer	1 (1.6)	
Vulvar cancer	6 (9.5)	
Anal cancer	7 (11.1)	
Cervical cancer	8 (12.7)	
Others	6 (9.5)	
Type of diagnosis		
Primary tumor	19 (30.2)	
Recurrent disease	44 (69.8)	
Albumin prep. (g/l)	59	40.6 (35.6-43.4)
Hb prep. (g/dl)	63	7.1 (6.6-7.7)
Neoadjuvant therapy		
No	22 (34.9)	
Yes	41 (65.1)	
Intraoperative data		
Operating time (min)	63	583.0 (494.0-695.0)
Blood loss (ml)	58	2150.0 (1200.0-3275.0)
Bone resection		
No	35 (55.6)	
Yes	28 (44.4)	
Pelvic closure		
Direct suturing	22 (34.9)	
VRAM	21 (33.3)	
Bioresorbable Mesh graft	8 (12.7)	
VRAM + Mesh graft	10 (15.9)	
Others	2 (3.2)	
Postoperative data		
Histological type		
Adenocarcinoma	40 (63.5)	
Squamous cell carcinoma	20 (31.7)	
Others	3 (4.8)	
Nodal status (pelvic exenteration)		
N0	48 (76.2)	
N1/2	15 (23.8)	
Metastasis at pelvic exenteration		
M0	56 (88.9)	
M1	7 (11.1)	
Resection status		
R0	41 (65.1)	
R1	12 (19.0)	
R2	1 (1.6)	
Rx	9 (14.3)	
Surgical complication		
No	17 (27.0)	
Yes	46 (73.0)	
Clavien-Dindo classification		
Grade 0	8 (12.7)	
Grade 1	6 (9.5)	
Grade 2	7 (11.1)	
Grade 3a	7 (11.1)	
Grade 3b	26 (41.3)	
Grade 4a	7 (11.1)	
Grade 4b	2 (3.2)	
Need for reoperation		
No	28 (44.4)	
Yes	35 (55.6)	
Surgical site infection perineal		
No	31 (49.2)	
Yes	32 (50.8)	
Medical complication		
No	49 (77.8)	
Yes	14 (22.2)	

*No.*, number of patients; *IQR*, interquartile range; *BMI*, body mass index; *ASA score*, American Society of Anesthesiology score; *PRBC*, packed red blood cells; *min*, minutes; *ml*, milliliter; *g/l*, gram per liter; *g/dl*, gram per deciliter; *Hb*, hemoglobin; *prep.*, preoperative; *VRAM*, vertical rectus abdominis musculocutaneous flap; *ICU*, intensive care unit

A total of 41 patients (65.1%) received neoadjuvant therapy (16 for primary tumors and 25 for recurrent disease; for detailed information, see also Supplementary Table 1). Out of 44 patients (27.3%) with recurrent disease, 10 were subjected exclusively to chemotherapy, 13 (29.5%) received radio-chemotherapy, and two patients received only radiotherapy. R0, R1, R2, and Rx resections were achieved in 65.1%, 19%, 1.6%, and 14.3% of the patients, respectively (Table 1).

### Postoperative complications and perioperative outcome of patients undergoing PE for colorectal and non-colorectal cancer

In our cohort, the 30-day mortality was 0%. The 90-day mortality rate was 1.6% (1 out of 63), which was due to multi-organ failure with a total in-hospital mortality of 3.2% (2 out of 63). Postoperative complications were classified according to Dindo et al. [14]. Grade 0 was observed in 8 patients (12.7%), grade 1 in 6 patients (9.5%), and grade 2 in 7 patients (11.1%). Seven patients needed additional interventional therapy (grade 3a; 11.1%) and 26 patients required a reoperation due to complications (grade 3b; 41.3%). In addition to reoperation, septic complications with failure of at least one organ system occurred in 7 patients (grade 4a; 11.1%) and multi-organ failure in two patients (grade 4b; 3.2%) (Supplementary Table 2). Surgical revision was mostly driven by surgical site perineal (25.0%) and abdominal (13.8%) infections with consecutive need for vacuum-based wound therapy and musculocutaneous flap reconstruction (10.0%). Relaparotomy was necessary in 15.0% (Supplementary Table 3).

On univariate analysis, only neoadjuvant treatment was found to be a risk factor for postoperative complications (Clavien-Dindo score ≥ 3b) (*P* = 0.005) (Table 2). In contrast, neither tumor entity (colorectal cancer vs. non-colorectal cancer) nor histological type (adenocarcinoma vs. squamous cell carcinoma vs. others) nor type of diagnosis (primary tumor vs. recurrent tumor) was significantly associated with the incidence of complications (Clavien-Dindo score ≥ 3b). Likewise, neither bone resection nor pelvic closure technique was a significant risk factor for postoperative complications (Table 2). Multivariate analysis confirmed the application of neoadjuvant therapy to be independently associated with perioperative complications (odds ratio 4.441; 95% confidence interval [CI]: 1.375–14.342, *P* = 0.013). A subgroup analysis revealed radiotherapy to be of higher likelihood for reoperation (Clavien-Dindo score ≥ 3b) (*P* = 0.005).

**Table 2:** Risk factors for complications (need for reoperation)

			Morbidity (Dindo > 3a)			
			Univariate	Multivariate		
Variables	No (no./mean)	Yes (no./mean)	*P* value	*P* value	OR	95% CI
Age	61.0	60.6	0.885	0.889	-	-
Gender			0.910	0.284	-	-
Male	14	17				
Female	14	18				
BMI	25.4	24.2	0.314	0.487	-	-
ASA			0.952	0.949	-	-
I/II	9	11				
III/IV	19	24				
Operating time (min)	557.1	630.5	0.056	0.133	-	-
Blood loss (ml)			0.210			
PRBC transfusion during operation			0.183			
Diagnosis			0.608			
Colorectal cancer	15	21				
Others	13	14				
Histological type			0.718			
Adenocarcinoma	17	23				
Squamous cell carcinoma	9	11				
Others	2	1				
Type of diagnosis			0.390			
Primary tumor	10	9				
Recurrent disease	18	26				
Albumin prep. g/l (35.0-52.0 g/l)	39.7	39.5	0.736			
Hb prep. g/dl (8.6-12.1 mmol/l)	7.13	7.21	0.736			
Neoadjuvant therapy			0.005	0.013	4.441	1.375-14.342
No	15	7				
Yes	13	28				
Nodal status			0.427			
N0	20	8				
N1/2	8	7				
Metastasis at operation			0.370			
M0	26	2				
M1	2	5				
Resection status			0.679			
R0	19	22				
R1/2/x	9	13				
Bone resection			0.678			
No	14	20				
Yes	13	15				
Pelvic closure			0.358			
Suturing/bioresorbable Mesh graft	15	15				
VRAM/VRAM + Mesh graft	13	18				

*No.*, number of patients; *OR*, odds ratio; *95% CI*, 95% confidence interval; *BMI*, body mass index; *ASA score*, American Society of Anesthesiology score; *PRBC*, packed red blood cells; *g/l*, gram per liter; *g/dl*, gram per deciliter; *min*, minutes; *ml*, milliliter; *Hb*, hemoglobin; *AJCC (TNM)*, American Joint Committee on Cancer Tumor Classification; *prep.*, preoperative; *VRAM*, vertical rectus abdominis musculocutaneous flap

### Oncological long-term outcome of patients undergoing PE for colorectal and non-colorectal cancer

#### Overall survival

##### Total cohort

**Fig. 1:**
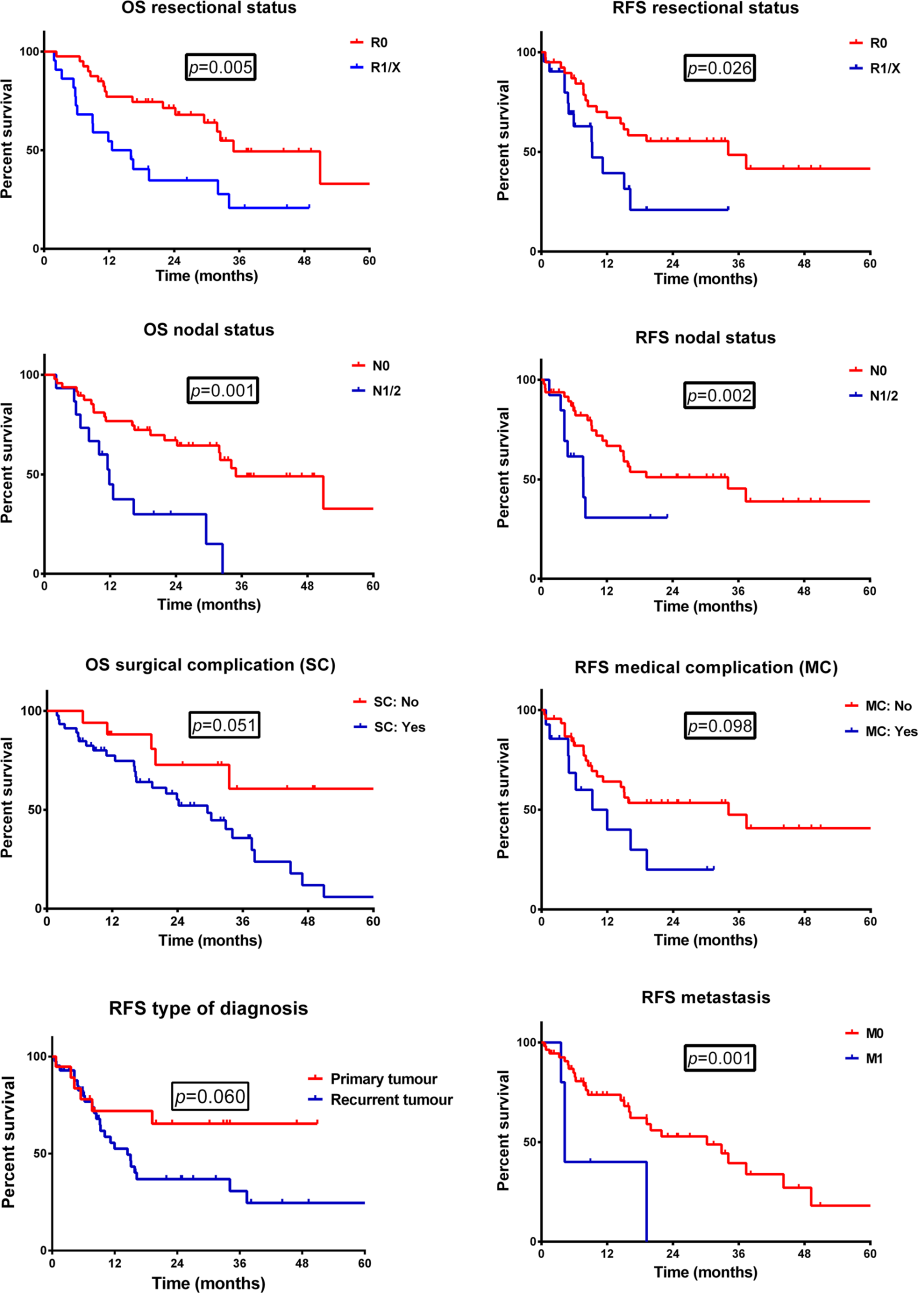
Prognostic effect on overall and recurrence-free survival. Survival analysis for clinical pre-, intra-, and postoperative variables for overall survival and recurrence-free survival (log-rank testing). OS, overall survival; RFS, recurrence-free survival

The median follow-up (FU) period and median overall survival for the whole group was 19.4 (IQR 10.0–32.9) months. Univariate analysis (log-rank test) revealed duration of operation (*P* = 0.013), preoperative hypoalbuminemia (*P* = 0.023), and the incidence of surgical (*P* = 0.05) and medical (*P* = 0.038) complications as perioperative risk factors for decreased overall survival (Table 3; Fig. 1; Supplementary Fig. 1). Among tumor-associated factors, metastatic status (M1) before operation (*P* = 0.022), positive nodal status (*P* = 0.001), and resection status (R1/2/x; *P* = 0.005) correlated with worse OS (Table 3; Fig. 1; Supplementary Fig. 2) in the whole cohort. It is noteworthy that the 2-year survival rate was 73.2% following R0 resection and 36.7% after R1/R2/Rx resection (*P* = 0.003) in the univariate analysis (Supplementary Table 5). No difference between CRC and other tumor entities was seen in OS (*P* = 0.532). Neoadjuvant therapy also did not show influence on OS (*P* = 0.521).

**Table 3:** Risk factors for survival

	Overall survival				Recurrence-free survival			
	Log-rank	Cox regression			Log-rank	Cox regression		
Variable	*P* value	*P* value	HR	95% CI	*P* value	*P* value	HR	95% CI
Age	0.159	0.021	1.044	1.006-1.084	0.914	0.479		
Gender	0.493	0.534			0.210	0.271		
Male								
Female								
BMI	0.423	0.432			0.639	0.564		
ASA	0.172	0.005	0.230	0.083-0.640	0.868	0.536		
I/II								
III/IV								
Operating time (min)	0.013	0.332			0.067	0.595		
Blood loss (ml)	0.565	0.028	1.000	1.000-1.001	0.760	0.234		
Diagnosis	0.532				0.976			
Colorectal cancer								
Others								
Histological type	0.419				0.217			
Adenocarcinoma								
Squamous cell carcinoma								
Others								
Type of diagnosis	0.408				0.060	0.002	8.452	2.209-32.338
Primary tumor								
Recurrent disease								
Albumin prep. g/l (35.0-52.0 g/l)	0.023	0.736			0.177	0.036	0.918	0.847-0.995
Hb prep. g/dl (8.6-12 g/dl)	0.147	< 0.001	0.261	0.137-0.498	0.726	0.612		
Neoadjuvant therapy	0.521				0.289			
No								
Yes								
Nodal status	0.001	< 0.001	12.999	4.218-40.055	0.002	0.003	5.560	1.817-18.858
N0								
N1/2								
Metastasis at operation	0.022	0.661			0.001	0.001	6.176	2.022-18.858
M0								
M1								
Resection status	0.005	0.681			0.026	0.712		
R0								
R1/2/x								
Bone resection	0.540				0.160			
No								
Yes								
Pelvic closure	0.623				0.641			
Direct suturing/bioresorbable Mesh graft								
VRAM/VRAM + Mesh graft								
Others								
Need for reoperation (Dindo > 3a)	0.085	0.177			0.983			
No								
Yes								
Surgical complication	0.051	0.003	5.125	1.751-14.996	0.139			
No								
Yes								
Surgical site infection perineal	0.145	0.169			0.466			
No								
Yes								
Medical complication	0.038	0.199			0.098	0.001	6.925	2.160-22.199
No								
Yes								

*No.*, number of patients; *IQR*, interquartile range; *HR*, hazard ratio; *95% CI*, 95% confidence interval; *BMI*, body mass index; *ASA score*, American Society of Anesthesiology score; *PRBC*, packed red blood cells; *g/l*, gram per liter; *g/dl*, gram per deciliter; *Hb*, hemoglobin; *AJCC (TNM)*, American Joint Committee on Cancer Tumor Classification; *prep.*, preoperative; *VRAM*, vertical rectus abdominis musculocutaneous flap

Multivariate analysis identified advanced age (hazard ratio [HR] 1.044; 95% CI: 1.006-1.084; *P* = 0.021), increased blood loss during operation (HR 1.000; 95% CI: 1.000-1.001; *P* = 0.028), positive nodal status (HR 12.999; 95% CI: 4.218-40.055; *P* = 0.028), and the incidence of postoperative complications (HR 12.999; 95% CI: 4.218-40.055; *P* = 0.003) as independent risk factors in the whole cohort. Patients with ASA status I/II (HR 0.230; 95% CI: 0.083-0.640; *P* = 0.005) and higher levels of preoperative hemoglobin (HR 0.261; 95% CI: 0.137-0.498; *P* < 0.001) showed better long-time survival (Table 3).

##### R0 subgroup

In the subgroup analysis for patients with an R0 resection, nodal status was identified to be a negative prognostic parameter for overall survival in the uni- (*P*= 0.003) and multivariate analysis (HR 59.205; 95% CI: 4.532-773.459; *P* = 0.002) (Supplementary Table 3). In contrast, neither tumor entity nor histological type was significantly associated with different median overall survival.

#### Recurrence-free survival

##### Total cohort

The recurrence-free survival for all patients reached 9.3 (IQR 5.0–24.7) months. The majority of the patients suffered from local recurrence (34.4%) or peritoneal carcinomatosis (12.5%). Metachronous metastases developed in the lung (9.4%), liver (6.3%), lymph nodes (6.3%), bones (3.1%), and bilaterally in the adrenal glands (3.1%). In the whole cohort, univariate analysis (log-rank test) identified nodal status (*P* = 0.003), presence of metastases at time of PE (*P* = 0.001), and resection status (*P* = 0.026) to be associated with early relapse, whereas tumor type (primary vs. recurrence) just missed the significance level (Table 3; Fig. 1). Multivariate analysis confirmed nodal status (HR 5.560; 95% CI: 1.817-18.858; *P* = 0.003) and presence of metastases at time of PE (HR 6.176; 95% CI: 2.022-18.858; *P* = 0.001) as independent risk factors for recurrent disease. Additionally, recurrent tumor type (HR 8.452; 95% CI: 2.209-32.338; *P* = 0.002), hypoalbuminemia (HR 0.918; 95% CI: 0.847-0.995; *P* = 0.036), and the occurrence of perioperative medical complications (HR 6.925; 95% CI: 2.160-22.199; *P* = 0.001) correlated significantly with a shortened recurrence-free survival. In contrast, resection status, tumor entity (colorectal vs. non-colorectal), and histological type failed to be significant risk factors in the multivariate analysis (Table 3; Supplementary Fig. 2).

##### R0 subgroup

The 2-year recurrence-free survival for R0-resected patients was 52.4%. The subgroup analysis for patients after R0 resection confirmed nodal status (*P* = 0.026) and presence of metastases at time of PE (*P* = 0.001) to correlate with a decreased recurrence-free survival in the univariate analysis. In the multivariate Cox model, only metastatic situation (HR 24.817; 95% CI: 3.291-187.128; *P* = 0.002) revealed level of significance.

## Discussion

The objective of our study was to investigate whether there are significant differences in the perioperative and long-term outcome of patients with colorectal cancer and non-colorectal cancer undergoing pelvic exenteration. We were able to show that perioperative mortality was relatively low regardless of whether patients were subjected to a PE for colorectal cancer or non-colorectal cancer. These results are in good accordance with previous studies reporting a 30-day mortality rate of between 0.0 and 8.7% for patients undergoing PE for rectal cancer [5]. However, PE has a significant risk of perioperative complications with reported rates between 31.6 and 86.0% [5, 15]. In our cohort, 73.0% of the patients had at least one postoperative surgical complication. This corresponds to the rate of complications that have also been reported in numerous other studies ranging between 37 and 100% [15, 16]. Most postoperative complications in our cohort were abscesses in the small pelvis, surgical site infection of the abdominal wound, and insufficiency of the fascial suture. These types of complications have been frequently reported in other studies for patients undergoing a PE. One of the most significant risk factors for the occurrence of postoperative complications is the administration of neoadjuvant therapy [6]. This parameter was also identified in our cohort as the only significant risk factor in the uni- and multivariate analysis. Especially radiotherapy-caused scar tissue complicates surgical preparation and increases the risk for reoperation, regarding surgical site infections. Neoadjuvant therapy might play a beneficial role in tumor downstaging and increasing the chance of negative resection margins. However, it is also important to take into account that many studies, including our own data, could not show an effect of neoadjuvant therapy on survival for either colorectal or non-colorectal cancer [6, 15]. Therefore, neoadjuvant therapy should only be considered if the probability of achieving an R0 resection can be increased. However, it is worthy of note that no significant association with postoperative complications was found for tumor entity (colorectal cancer vs. non-colorectal cancer), histological type, or the type of diagnosis (primary tumor vs. recurrent tumor). Moreover, when bone resection was performed to achieve a clear margin, it was not associated with an increase in postoperative complications. These data confirm previous studies indicating that there are no relevant contraindications for pelvic exenteration when the sacrum is involved [17]. In contrast, neither preoperative anemia nor preoperative hypoalbuminemia was found to be a predictive factor for postoperative morbidity. This is in line with recent reports where pelvic exenteration was performed for recurrent gynecological malignancies [18, 19].

With regard to factors that determine the long-term outcome of patients undergoing a PE, advanced age as well as an ASA III status was associated with an elevated risk of lower OS but not RFS. These variables indicate an increased risk of death for patients with comorbidities after major high morbidity surgery and should be discussed with the patients prior to the surgical procedure. Furthermore, we have evaluated factors that play a pivotal role in the preoperative and perioperative management of patients undergoing a PE. Survival analysis revealed that high preoperative hemoglobin levels were predictive of better OS and patients with higher serum albumin levels showed better RFS. These results underscore the need for better preoperative conditioning to normalize patients’ hemoglobin levels (blood management) by giving iron infusions and other supplements, thereby reducing the need for blood transfusion in perioperative settings [20]. A high preoperative serum protein level is necessary for improving patients’ nutritional status. It is therefore important to ensure adequate caloric intake, either orally or intravenously [21]. All these perioperative measures are aligned with ERAS programs, which include numerous methods for improving preoperative health, postoperative mobilization, and digestion in patients, especially those with a long history of cancer. The earlier conditioning of the patient is begun, the better the outcome may be. This leaves room for future trials [13, 22].

With regard to tumor-associated factors, we show that a positive nodal status and positive resection margins are strong predictors for decreased OS and RFS, which is in good accordance with previous studies [6, 15]. Moreover, we could verify that primary tumors have better OS and RFS than recurrent cancer [23]. In addition, our data of the subgroup analysis suggest that a limited metastatic situation like singular metastasis in the liver and lung is not significantly associated with OS if an R0 resection is possible. However, as an indication of earlier tumor relapse, it might be of interest for future studies in order to extend the surgical indication. Therefore, individual approaches should be discussed in interdisciplinary boards and with the patient. We identified the presence of surgical complications and high blood loss during operation as independent factors influencing OS. This indicates that high morbidity in complicated operations in patients with comorbidities may lead to satisfactory short-term results. Given their weakened state, however, these patients are often susceptible to death from causes other than cancer [24].

## Conclusion

Pelvic exenteration in R0-resected patients reaches a 2-year OS and RFS of 73.2% and 52.4%, respectively, in a very challenging patient cohort. However, this is a complex procedure involving multiple disciplines (general, vascular, and plastic surgery, urology, anesthesiology, intensive care medicine, rehabilitation therapy, etc.). Consequently, there is a strong need for centralizing and operating these patients in high-volume hospitals with high expertise and a standardized management. These are the prerequisites to achieve low morbidity (e.g., lower blood loss), a high likelihood of achieving an R0 status, and the development of novel surgical concepts such as minimally invasive surgery techniques in pelvic exenteration [25]. If these conditions are met, pelvic exenteration is a safe procedure for different types of tumor entities and offers the best chance of cure for locally advanced primary or recurrent pelvic organ malignancies invading adjacent organs.

## Supplementary information

ESM 1 (DOCX 39 kb)

ESM 2 (PNG 64 kb)

High resolution Image (TIF 725 kb)

ESM 3 (PNG 55 kb)

High resolution Image (TIF 607 kb)

## Data Availability

All authors declare that they had full access to all information, data, software, and codes used for and published in this article. These data have not been published elsewhere.
