# Supplementary material for: Pelvic exenteration for colorectal and non-colorectal cancer: a comparison of perioperative and oncological outcome
Source: Int J Colorectal Dis. 2021 Mar 7;36(8):1701–10. doi: 10.1007/s00384-021-03893-y (PMC8279979; doi:10.1007/s00384-021-03893-y)
Supplement: Supplementary file 1 — (DOCX 39 kb) [file 384_2021_3893_MOESM1_ESM.docx]

**Supplementary data:**

**Table S1: neoadjuvant therapy**

| **Primary Tumour (n=19)** | **Chemotherapy** | **Radiotherapy** | **Radio-Chemotherapy** |
| --- | --- | --- | --- |
| **Colorectal (n=10)** | **1** | **1** | **8** |
| **Vulvic (n=0)** | **0** | **0** | **0** |
| **Anal (n=3)** | **0** | **0** | **3** |
| **Cervical (n=2)** | **0** | **0** | **0** |
| **Others (n=4)** | **0** | **1** | **1** |
| **Recurrent Tumour (n=44)** |  |  |  |
| **Colorectal (n=26)** | **6** | **0** | **12** |
| **Vulvic (n=6)** | **0** | **1** | **1** |
| **Anal (n=4)** | **0** | **0** | **0** |
| **Cervical (n=6)** | **4** | **1** | **0** |
| **Others (n=2)** | **0** | **0** | **0** |

**Table S2: Complications during hospital course**

| **Surgical complications (46/63 patients; 73.0%)** | **Number (%)** |
| --- | --- |
| Abscess formation in the pelvis | 14 (21.9) |
| Surgical site infection abdominal wound | 11 (17.2) |
| Burst abdomen | 7 (10.9) |
| Leakage of the gut | 6 (9.4) |
| Postoperative bleeding | 5 (7.8) |
| Complications ileac conduit | 5 (7.8) |
| Stoma complications | 4 (6.3) |
| Venous port infection | 3 (4.7) |
| Mechanical ileus | 2 (3.1) |
| Others | 7 (10.9) |
| **Medical complications (14/63 patients; 22.2%)** | **Number (%)** |
| Respiratory decompensation (pneumonia) | 3 (21.4) |
| Renal insufficiency | 1 (7.1) |
| Cardiac decompensation (arrhythmia, infarction) | 2 (14.3) |
| Stroke/transient ischemic attack | 2 (14.3) |
| Catheter sepsis | 2 (14.3) |
| Pulmonary embolism | 2 (14.3) |
| Deep venous thrombosis | 2 (14.3) |

**Table S3: Operational procedures in need for reoperation**

| **Surgical procedures** | **Number (%)** |
| --- | --- |
| Debridement abdominal wall + vacuum therapy | 11 (13,8) |
| Venous port explantation | 5 (6,3) |
| Relaparotomy | 12 (15,0) |
| Small bowel resection | 4 (5,0) |
| Musculo-cutaneous flap reconstruction | 8 (10,0) |
| Stoma revision | 5 (6,3) |
| Debridement perineal + vacuum therapy | 20 (25,0) |
| Excision fistula | 1 (1.3) |
| Arterio-venous loop | 1 (1.3) |
| Femoro-femoro cross over bypass | 1 (1.3) |
| Fascia incision lower leg | 1 (1.3) |
| Embolectomy and stent implantation femoral | 1 (1.3) |
| Colectomy | 1 (1.3) |
| **Urological precedures** |  |
| Percutaneous nephrostomy | 3 (3,8) |
| Reimplantation ureter | 2 (2,5) |
| Change of ureter catheter | 4 (5,0) |

**Table S4:** Risk factors for survival in the subgroup of patients with R0 resection

|  | **Overall survival** | | | | **Recurrence free survival** | | | |
| --- | --- | --- | --- | --- | --- | --- | --- | --- |
|  | **Log-rank** | **Cox - regression** | | | **Log-rank** | **Cox - regression** | | |
| **Variable** | **P-value** | **P-value** | **HR** | **95%CI** | **P-value** | **P-value** | **HR** | **95%CI** |
| **age** | 0.223 | **0.086** |  |  | 0.438 | 0.224 |  |  |
| **gender** | 0.915 | **0.007** | **0.037** | **0.003-0.407** | 0.747 | 0.174 |  |  |
| male |  |  |  |  |  |  |  |  |
| female |  |  |  |  |  |  |  |  |
| **BMI** | 0.072 | **0.002** | **0.681** | **0.536-0.866** | 0.047 | 0.124 |  |  |
| **ASA** | 0.742 | **0.001** | **0.003** | **0.000-0.094** | 0.660 | 0.033 | 0.212 | 0.051-0.883 |
| I/II |  |  |  |  |  |  |  |  |
| III/IV |  |  |  |  |  |  |  |  |
| **operating time (min)** | 0.312 | **0.003** | **1.019** | **1.007-1.032** | **0.028** | **0.001** | **1.014** | **1.006-1.021** |
| **blood loss (ml)** | 0.395 | **0.015** | **0.999** | **0.998-1.000** | 0.662 | **0.011** | **0.999** | **0.998-1.000** |
| **Diagnosis** | 0.442 |  |  |  | 0.765 |  |  |  |
| Colorectal cancer |  |  |  |  |  |  |  |  |
| Others |  |  |  |  |  |  |  |  |
| **Histological Type** | 0.749 |  |  |  | 0.636 |  |  |  |
| Adeno carcinoma |  |  |  |  |  |  |  |  |
| Squamous-cell carcinoma |  |  |  |  |  |  |  |  |
| Others |  |  |  |  |  |  |  |  |
| **Type of diagnosis** | 0.627 |  |  |  | 0.572 |  |  |  |
| Primary Tumor |  |  |  |  |  |  |  |  |
| Recurrent disease |  |  |  |  |  |  |  |  |
| **Albumin prep. g/l (35.0-52.0g/l)** | 0.200 |  |  |  | 0.265 | 0.985 |  |  |
| **Hb prep. g/dl (8.6-12.g/dl)** | 0.210 | 0.358 |  |  | 0.725 | 0.497 |  |  |
| **neoadjuvant therapy** | 0.534 |  |  |  | 0.810 |  |  |  |
| No |  |  |  |  |  |  |  |  |
| Yes |  |  |  |  |  |  |  |  |
| **Nodal status** | **0.003** | **0.002** | 59.205 | **4.532-773.459** | **0.026** | 0.153 |  |  |
| N0 |  |  |  |  |  |  |  |  |
| N1/2 |  |  |  |  |  |  |  |  |
| **Metastasis at operation** | 0.105 | 0.413 |  |  | **<0.001** | **0.002** | **24.817** | **3.291-187.128** |
| M0 |  |  |  |  |  |  |  |  |
| M1 |  |  |  |  |  |  |  |  |
| **Partial sacrectomy** | 0.880 |  |  |  | 0.156 |  |  |  |
| No |  |  |  |  |  |  |  |  |
| Yes |  |  |  |  |  |  |  |  |
| **Pelvic closure** | 0.386 |  |  |  | 0.583 |  |  |  |
| Direct suturing/bioresorbable Meshgraft |  |  |  |  |  |  |  |  |
| VRAM/VRAM + Meshgraft |  |  |  |  |  |  |  |  |
| Others |  |  |  |  |  |  |  |  |
| **Need for Reoperation (Dindo >3a)** | 0.148 |  |  |  | 0.867 |  |  |  |
| No |  |  |  |  |  |  |  |  |
| Yes |  |  |  |  |  |  |  |  |
| **Surgical complication** | 0.155 |  |  |  | 0.166 |  |  |  |
| No |  |  |  |  |  |  |  |  |
| Yes |  |  |  |  |  |  |  |  |
| **Surgical site infection perineal** | 0.300 |  |  |  | 0.526 |  |  |  |
| No |  |  |  |  |  |  |  |  |
| Yes |  |  |  |  |  |  |  |  |
| **Medical complication** | 0.286 |  |  |  | 0.380 |  |  |  |
| No |  |  |  |  |  |  |  |  |
| Yes |  |  |  |  |  |  |  |  |

**Table S5: risk factors for 2 – year survival**

|  | **2y-Overall survival** | | | | **2y-Recurrence free survival** | | | |
| --- | --- | --- | --- | --- | --- | --- | --- | --- |
|  | **Log-rank** | **Cox - regression** | | | **Log-rank** | **Cox - regression** | | |
| **Variable** | **P-value** | **P-value** | **HR** | **95%CI** | **P-value** | **P-value** | **HR** | **95%CI** |
| **age** | 0.182 | **0.008** | **1.068** | **1.017-1.122** | 0.827 | 0.479 |  |  |
| **gender** | 0.504 | 0.384 |  |  | 0.251 | 0.271 |  |  |
| male |  |  |  |  |  |  |  |  |
| female |  |  |  |  |  |  |  |  |
| **BMI** | 0.726 | 0.969 |  |  | 0.608 | 0.564 |  |  |
| **ASA** | 0.340 | **0.019** | **0.203** | **0.054-0.768** | 0.937 | 0.536 |  |  |
| I/II |  |  |  |  |  |  |  |  |
| III/IV |  |  |  |  |  |  |  |  |
| **operating time (min)** | **0.028** | 0.893 |  |  | **0.038** | 0.595 |  |  |
| **blood loss (ml)** | 0.636 | **0.003** | **1.001** | **1.000-1.001** | 0.979 | 0.234 |  |  |
| **Diagnosis** | 0.954 |  |  |  | 0.870 |  |  |  |
| Colorectal cancer |  |  |  |  |  |  |  |  |
| Others |  |  |  |  |  |  |  |  |
| **Histological Type** | 0.296 |  |  |  | 0.214 |  |  |  |
| Adeno carcinoma |  |  |  |  |  |  |  |  |
| Squamous-cell carcinoma |  |  |  |  |  |  |  |  |
| Others |  |  |  |  |  |  |  |  |
| **Type of diagnosis** | 0.065 | **<0.001** | **47.369** | **5.730-391.578** | **0.089** | **0.019** | **3.627** | **1.232-10.677** |
| Primary Tumor |  |  |  |  |  |  |  |  |
| Recurrent disease |  |  |  |  |  |  |  |  |
| **Albumin prep. g/l (35.0-52.0g/l)** | **0.075** | 0.735 |  |  | 0.177 | **0.036** |  |  |
| **Hb prep. g/dl (8.6-12.g/dl)** | 0.122 | **<0.001** | **0.152** | **0.061-0.381** | 0.905 | 0.612 |  |  |
| **neoadjuvant therapy** | 0.819 |  |  |  | 0.480 |  |  |  |
| No |  |  |  |  |  |  |  |  |
| Yes |  |  |  |  |  |  |  |  |
| **Nodal status** | **0.007** | **<0.001** | **22.014** | **5.420-89.407** | **0.002** | **0.003** | 4.590 | **1.653-12.748** |
| N0 |  |  |  |  |  |  |  |  |
| N1/2 |  |  |  |  |  |  |  |  |
| **Metastasis at operation** | 0.246 |  |  |  | **0.001** | **0.013** | **3.874** | **1.323-11.345** |
| M0 |  |  |  |  |  |  |  |  |
| M1 |  |  |  |  |  |  |  |  |
| **Resection status** | **0.003** | 0.718 |  |  | **0.022** | 0.069 | 2.231 | 0.941-5.292 |
| R0 |  |  |  |  |  |  |  |  |
| R1/2/X |  |  |  |  |  |  |  |  |
| **Bone resection** | 0.986 |  |  |  | 0.103 |  |  |  |
| No |  |  |  |  |  |  |  |  |
| Yes |  |  |  |  |  |  |  |  |
| **Pelvic closure** | 0.541 |  |  |  | 0.377 |  |  |  |
| Direct suturing/bioresorbable Mesh graft |  |  |  |  |  |  |  |  |
| VRAM/VRAM + Mesh graft |  |  |  |  |  |  |  |  |
| **Need for Reoperation (Dindo >3a)** | 0.102 |  |  |  | 0.871 |  |  |  |
| No |  |  |  |  |  |  |  |  |
| Yes |  |  |  |  |  |  |  |  |
| **Surgical complication** | **0.043** | **0.002** | **10.331** | **2.323-45.946** | 0.217 |  |  |  |
| No |  |  |  |  |  |  |  |  |
| Yes |  |  |  |  |  |  |  |  |
| **Surgical site infection perineal** | 0.224 |  |  |  | 0.424 |  |  |  |
| No |  |  |  |  |  |  |  |  |
| Yes |  |  |  |  |  |  |  |  |
| **Medical complication** | 0.523 |  |  |  | 0.098 | **0.001** | **6.925** | **2.160-22.199** |
| No |  |  |  |  |  |  |  |  |
| Yes |  |  |  |  |  |  |  |  |
