# Supplementary figures and images for: Pelvic exenteration for colorectal and non-colorectal cancer: a comparison of perioperative and oncological outcome
Source: Int J Colorectal Dis. 2021 Mar 7;36(8):1701–10. doi: 10.1007/s00384-021-03893-y (PMC8279979; doi:10.1007/s00384-021-03893-y)

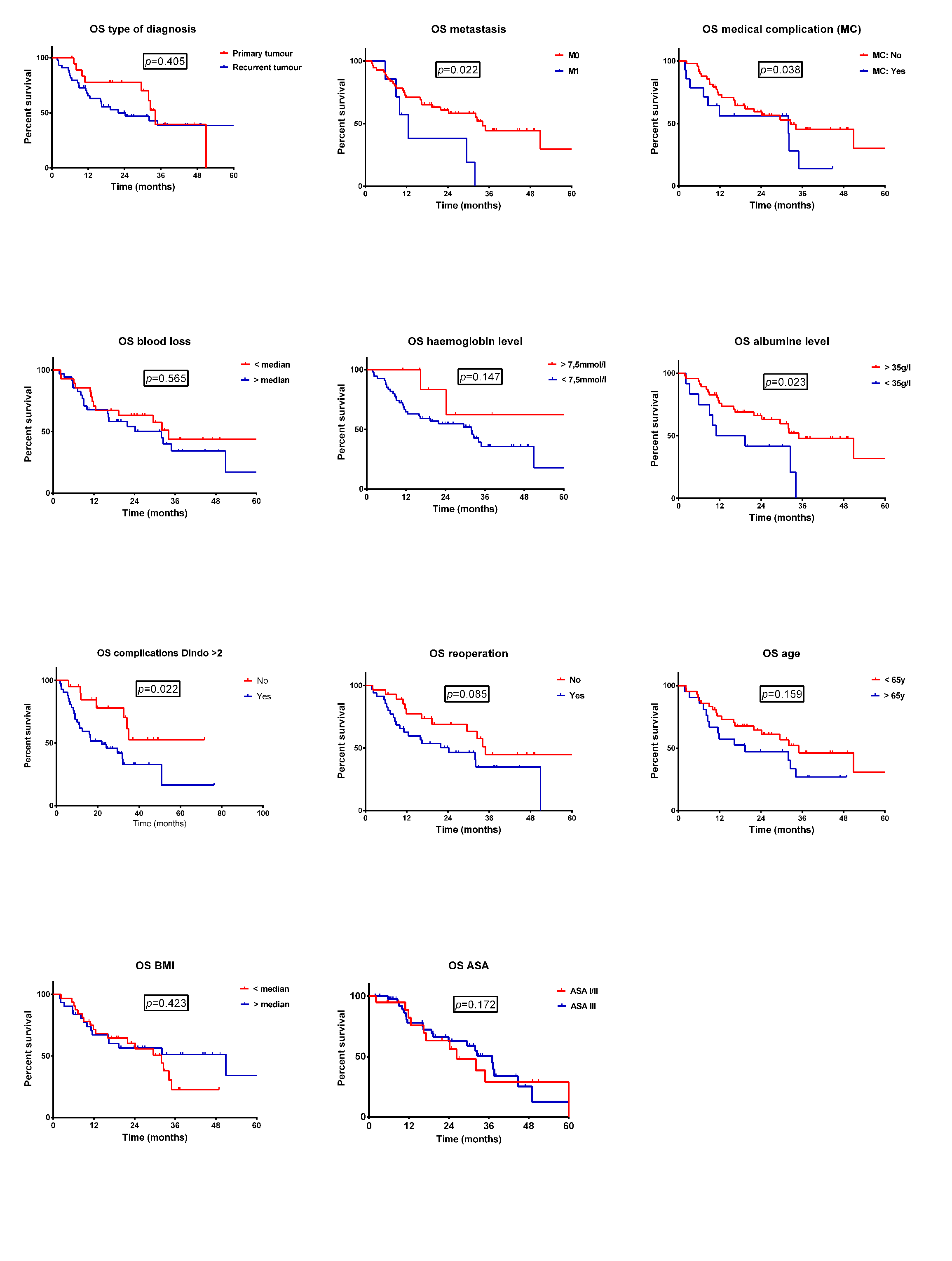

Supplement: Supplementary file 2 — (PNG 64 kb) [file 384_2021_3893_Fig2_ESM.png]

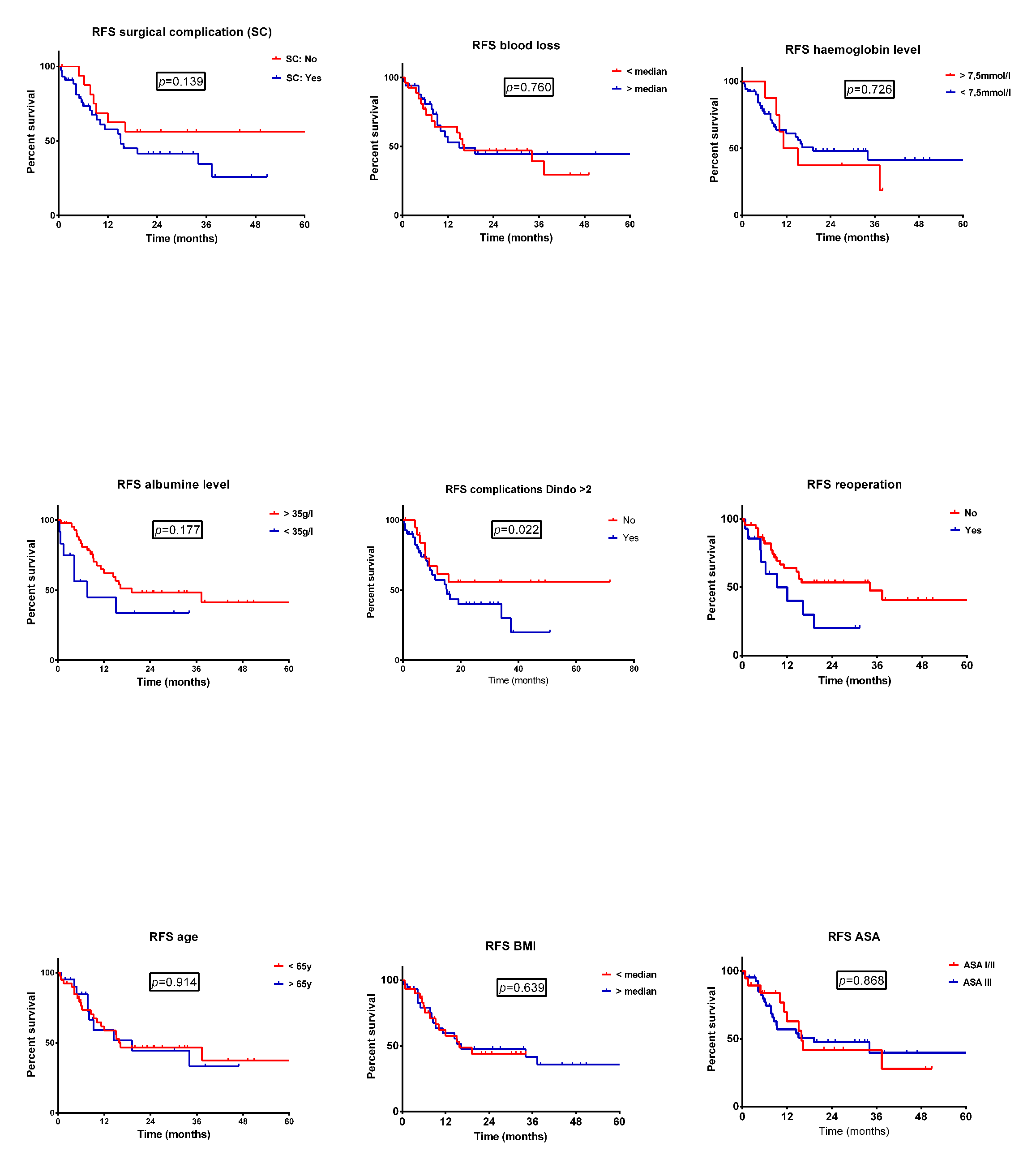

Supplement: Supplementary file 4 — (PNG 55 kb) [file 384_2021_3893_Fig3_ESM.png]
